# Change in Work–Time Control and Work–Home Interference Among Swedish Working Men and Women: Findings from the SLOSH Cohort Study

**DOI:** 10.1007/s12529-016-9565-8

**Published:** 2016-04-28

**Authors:** Constanze Leineweber, Göran Kecklund, Petra Lindfors, Linda L. Magnusson Hanson

**Affiliations:** 1 Stress Research Institute, Stockholm University, SE-106 91 Stockholm, Sweden; 2 Behavioural Science Institute, Radboud University, Nijmegen, The Netherlands; 3 Department of Psychology, Stockholm University, Stockholm, Sweden

**Keywords:** Gender, Work–home interference, Work–family conflict, Work–time control

## Abstract

**Purpose:**

The aim is to study the influence of change in work–time control (WTC) on work–home interference (WHI) while adjusting for other work-related factors, demographics, changes at work and WHI at baseline among women and men. An additional aim was to explore sex differences in the relation between change in WTC and WHI.

**Methods:**

The study included working participants of the Swedish Longitudinal Occupational Survey of Health (SLOSH) study of the third (2010) and fourth (2012) waves (*n* = 5440). Based on a seven-item index, four groups of WTC were formed: stable high (40 %), stable low (42 %), increasing (9 %), or decreasing (9 %) WTC over the 2 years. WHI was measured by four items and individuals were categorised in whether suffering or not suffering of WHI. Sex-stratified logistic regression analyses with 95 % confidence intervals (CI) were used to estimate the odds of experiencing WHI by change in WTC.

**Results:**

Controlling for demographics and work-related factors, women with stable low (OR = 1.46; 95 % CI 1.14–1.88) and women and men with decreasing WTC (women OR = 1.99; 95 % CI 1.38–2.85; men OR = 1.80; 95 % CI 1.18–2.73) had higher odds of WHI than those with a stable high WTC. Additionally, adjusting for changes at work and WHI at baseline did not alter the results substantially. Interaction analysis did not reveal any significant sex difference in the relation between WTC and WHI.

**Conclusions:**

For both women and men decreased and for women only, low control over working hours resulted in WHI also after adjusting for work-related factors and demographics.

## Introduction

Many employees experience difficulties in satisfactorily combining work and home demands, which may result in conflicts between these demands. In Sweden, more than 30 % of all parents seldom or never experience balance between demands from the work and home spheres [1], with figures from other European countries being similar. This type of imbalance may be associated with short- and long-term stress reactions and has been related to negative health outcomes including e.g. suboptimal sleep quality [2], poor self-rated health [2] and sickness absence [3]. Indeed, the European Agency for Safety and Health at Work Research has identified poor work–life balance as one of the top emerging psychosocial risks and warn for the negative consequences for the work force [4]. Thus, an adequate balance between work and private demands is important not only for the individual but also for organisations that want healthy and productive employees.

For organisations, one possible measure to facilitate the compatibility of work and home demands is to increase work–time control (WTC) which can be defined as ‘an employee’s possibilities of control over the duration, position and distribution of his or her work time’ [5]. Two factors of work–time control can be differentiated, i.e. ‘control over daily hours’ and ‘control over time off’ [6]. This employee-friendly flexibility is to be distinguished from employer-friendly flexibility [7], also labelled ‘variability’ in some papers [8], which refers to ‘the need of employers to extend, modify, or reduce hours according to client or production needs’ [5].

Flexible working hours may potentially have positive consequences for both employers and employees [9]. Today, organisations increasingly offer flexible work–time arrangements [10], e.g. staggered working hours, flexitime arrangement and work–time banking to increase employees’ possibilities to successfully combine home and work demands [11]. As the possibility to influence working times directly relate to the planning of time off work, WTC should have a positive effect on the possibility to combine work and non-work demands and make it easier to give priority to social and family needs. Indeed, several studies have shown positive associations between possibilities to influence working time and perceived work–home interference (WHI). Investigating a sample of human service employees in Germany, Kattenbach et al. found that time–autonomy reduced exhaustion and work–non-work conflict [12]. A Dutch study reported that time–spatial flexibility, i.e. part-time work, flexitime and telework from home, affected work–life balance positively [7]. Still, another study found that control over work time moderated the relationship between working hours and work–family balance, i.e. among employees with longer working hours, work–life balance was lower for workers with low WTC but not among those with higher WTC [13]. A systematic review concludes that there is relatively strong support to consider WTC as a promising tool for the maintenance of employees’ work–non-work balance [14]. However, this conclusion is based mainly on studies with cross-sectional designs, while longitudinal studies investigating the effects of WTC on WHI are scarce.

There are a number of factors which possibly could modify the relation between WTC and work–life balance. Having children living at home and marital status have been linked both to WTC [6] and WHI [15] and have also been shown to moderate the effect of schedule flexibility on WHI [16]. Also, socio-economic status, in terms of educational level, is clearly related to WTC [6], whereas findings regarding WHI are less consistent [15]. Still, it seems that, particularly among women, higher social position is associated with an increased risk for WHI [17]. Overtime and long working hours have been related to increased WTC [6] with several studies having confirmed that long working hours are associated with higher levels of WHI [18]. Finally, perceived control over work hours is strongly associated with job control in the demand–control model [19, 20], and work demands and work control are potential antecedents of WHI [21].

As regards women and men, it is unclear whether both groups benefit equally from having increased control over their working hours. It has been suggested that work–time flexibility may have adverse consequences for women’s work–home balance, as women may end up engaging in more non-work responsibilities [22]. This may, in turn, result in an increased total workload for women. However, empirical support for this hypothesis is limited. Whereas one study found that flexible work hours were beneficial for men only [23], another found that alternative work time arrangements (i.e. flexible work hours, job-sharing, telecommuting) decreased women’s work–life balance, while it had no influence on the work–life balance of men [22]. Yet, another study found that while women used their control over working hours to achieve a better work–life balance, men used flexible work schedules to increase their work commitment, thereby increasing their perceived work–family conflict [24]. Thus, existing findings are mixed and allow no clear conclusions concerning sex differences.

Moreover, results of WTC-related intervention studies have been mixed [25–27]. One intervention study of female nurses found positive changes for work–life balance, social support and job satisfaction after introducing self-scheduling [25]. However, another intervention study including female eldercare workers found no effects of increased WTC on work–life balance [27]. Yet, another intervention study found that changes in schedule control had similar positive effects on work–family conflict for men and women [26].

Taken together, further research is needed to delineate whether WTC will provide both women and men with adequate possibilities to combine work and private life demands and to clarify whether increased WTC is a good strategy for enhancing work life balance for both sexes.

This study aims to expand the understanding of the role of WTC for WHI by using an observational design with longitudinal data focusing on differences between women and men. Specifically, the study aimed at investigating how changes in WTC relate to WHI while also accounting for other work-related factors, family characteristics and changes in work situation in both women and men.

## Methods

### Study Sample

The study population consisted of the participants of the Swedish Longitudinal Occupational Survey of Health (SLOSH) study, a longitudinal cohort survey with a focus on the association between work organisation, work environment and health. The SLOSH sample is drawn from respondents to the Swedish Work Environment Surveys (SWES) conducted biennially by Statistics Sweden. In 2006, SLOSH started with a first follow-up of SWES participants 2003. In 2008, SWES 2005 participants were added to the cohort, totalling a population of 18,915 individuals.

The participants are followed by means of a postal questionnaire in two versions, one for those currently in paid work, i.e. working for at least 30 % of full time or a version for those not in paid work, i.e. those working less than 30 % or who are permanently or temporarily outside the labour force. The current paper included participants, who responded to the questionnaire for those in paid work (including self-employed) both in 2010 (wave 3) and 2012 (wave 4) (*n* = 5569). Of the 9131 working participants in 2010, 2818 (30.9 %) did not answer any questionnaire in 2012. A further 745 (25.4 %) completed the questionnaire for non-working participants. After exclusion of participants with missing values on work–time control at any time, the sample size was reduced to 5440 participants. Slightly more women and participants in a higher socio-economic group completed the questionnaires for working individuals in both 2010 and 2012. There were no age differences between respondents and non-respondents. Informed consent was obtained from all individual participants included in the study. Both SLOSH and the present study have been approved by the Regional Research Ethics Board in Stockholm.

### Measures

*WHI* was measured by an adapted version of a questionnaire developed by Fisher [28], which was designed to measure directions and domains of WHI. Four questions cover WHI (Cronbach’s alpha 0.89). Response alternatives ranged from 1 (‘not at all’) to 5 (‘nearly all the time’). Table 1 presents these questions and response frequencies. When at least three out of the four items were completed, a mean score was calculated. A mean of 3.5 or higher was considered to reflect WHI. This cut-off has satisfactory psychometric properties (specificity = 88 %, sensitivity = 77 %, kappa = 0.497) when compared with a single-item question ‘Do the demands placed on you at work interfere with your home and family life?’ which was dichotomised according to the response options ‘very often’ or ‘the whole time’, and used as a validity index.

**Table 1:** Items for work–home interference (WHI) measured in 2012 and corresponding frequencies for women and men

Item	Response alternative									
	‘Not at all’		‘Rarely’		‘Sometimes’		‘Often’		‘Nearly all time’	
	Men, *n* (%)	Women, *n* (%)	Men, *n* (%)	Women, *n* (%)	Men, *n* (%)	Women, *n* (%)	Men, *n* (%)	Women, *n* (%)	Men, *n* (%)	Women, *n* (%)
I come home from work too tired to do things I would like to do.	165 (7.05)	155 (5.10)	512 (21.87)	432 (14.21)	1086 (46.39)	1302 (42.81)	496 (21.19)	874 (28.74)	82 (3.50)	278 (9.14)
My job makes it difficult to maintain the kind of personal life I would like.	644 (27.50)	887 (29.19)	719 (30.70)	746 (24.55)	627 (26.77)	810 (26.65)	286 (12.21)	437 (14.38)	66 (2.82)	159 (5.23)
I often neglect my personal needs because of the demands of my work.	518 (22.12)	690 (22.73)	845 (36.08)	942 (31.04)	638 (27.24)	830 (27.35)	297 (12.68)	430 (14.17)	44 (1.88)	143 (4.71)
My personal life suffers because of my work.	612 (26.13)	834 (27.42)	806 (34.42)	935 (30.74)	636 (27.16)	829 (27.25)	238 (10.16)	336 (11.05)	50 (2.13)	108 (3.55)

*WTC* was measured by an adapted six-item Swedish version of the seven-item index developed by Ala-Mursula [20]. The items cover influence over scheduling and aspects of flexi-time, breaks, short time leave, vacation and the possibility to do private errands during working time. Items were combined and the composite score re-categorised into five response categories ranging from 1 = ‘very little’ to 5 = ‘very much’ WTC on average. Cronbach’s alpha was satisfying with a value of .88 in both 2010 and 2012. Four groups were constructed with regard to change in WTC (Table 2). Those who reported having ‘quite a little’ or very little control at both measurement times were considered to have *stable low* WTC (*n* = 2281, 41.9 %). Those who reported having WTC at least ‘to some extent’ at both measurement times were considered to have *stable high* WTC (*n* = 2202, 40.5 %). Further, those who reported very little or quite a little WTC at t1 but reported having WTC to at least ‘some extent’ at t2 were considered to have experienced an *increase* in WTC (*n* = 482, 8.9 %). Last, those who experienced WTC to some extent or more at t1 and quite a little or less WTC at t2 were considered as reporting *decreased* WTC (*n* = 475, 8.7 %).

**Table 2:** Number of respondents in four work–time control (WTC) categories (middle grey = stable low WTC, light grey = increasing WTC, dark grey = decreasing WTC, white = stable high WTC)

2012→ 2010↓	Very little	Quite a little	To some extent	Quite a bit	Very much
Very little	720	307	54	9	1
Quite a little	314	940	372	39	7
To some extent	50	359	967	231	42
Quite a bit	9	44	292	371	81
Very much	5	8	31	78	109

Sex, obtained from register data linked to questionnaire responses by means of the unique ten-digit personal identification numbers in Sweden, was used as a stratifying variable. Marital status, having children living at home, socio-economic status, work demands and decision authority, shift work and weekly working hours measured in 2010 were considered as possible confounders. *Marital status* was obtained by a single question with response alternatives ‘single’ or ‘married/cohabiting’ coded. *Having children living at home* was measured by a single question ‘Do you have any children living at home? *Include children living with you at least half of the time*.’ and coded into yes and no. The *socio-economic status* was based on the Swedish socio-economic classification (SEI) which not only builds mainly on the occupation (as reported in the questionnaire) but also takes length of education into account [29]. We used six categories, i.e. unskilled manual workers (reference category) [1], skilled manual workers [2], assistant non-manual employees [3], intermediate non-manual employees [4], professionals and upper-level executives [5], self-employed [6] and others. *Weekly working hours* was measured by one item with eight response categories, ranging from 1 = ‘<35 h/week’ to 8 = ‘>65 h/week’. In analyses, working hours were handled as a continuous variable. *Job demands* (five items) and *decision authority* (six items) were measured according to the demand–control model [30] and handled as continuous variables. *Shift work* was measured by a single item with nine response alternatives and recoded into six categories of shift work, covering daytime (forming the reference category), afternoon and evening work, shift work (two and three shift), roster work (which is defined as a more irregular type of shift work, e.g. common in the transport sector) and non-regulated working hours. A last alternative covered ‘other work time’. Further, we considered whether changes at work during the study period (i.e. 2010–2012) would explain possible changes in WTC and WHI. A better job position may be associated with more WTC but may also (due to higher demands) be associated with more WHI. Thus, having been promoted during the past 2 years and having got a new manager during the past 2 years, as measured in 2012, were included as potential explanatory variables (confounders) to changes in WTC.

### Statistical Analyses

Chi-square and analyses of variance tests were performed to evaluate differences in covariates and WHI between WTC categories. Sex-stratified logistic regression analyses were used to estimate how change in WTC was associated with odds of experiencing WHI and 95 % confidence intervals (CI). In the first step, we assessed the influence of change in WTC on WHI (Crude Model). In the next step (Model 1), we added demographic variables (i.e. marital status, having children living at home and socio-economic status) into the model. In the third step (Model 2), we added job demands, decision authority, working hours and shift work. In the fourth step (Model 3), we controlled for changes at work (i.e. promotion and new manager). Finally, in the last model (Model 4), we controlled for work home interference at baseline. To test if changes in WTC affected women and men differently, we calculated a synergy index with 95 % confidence intervals as a measure of additive interaction [31]. All analyses were performed in SAS 9.3.

## Results

Descriptive statistics are presented in Table 3. Slightly more women than men participated in the study. The mean age was about 49.6 years. Thirty-eight per cent of the participants worked between 36 and 40 h, and 30 % worked between 41 and 45 h/week. More than one quarter felt that they often or very often were too tired to do things they would like to do when they came home, but only around 13 % felt that their personal life suffered because of their work (Table 1). WHI decrease over time; in 2010, about 23 % experienced WHI, and in 2012, the percentage had decreased to 18.7 %. Generally, women reported WHI to a higher extent than men did. Also, clear differences in WHI between the four WTC groups were found.

**Table 3:** Means and standard deviations (SD) of work–home interference (WHI) and covariates 2010, also subdivided by change in work–time control (WTC)

	All	Stable high WTC *n* = 2202 (40.5 %)	Stable low WTC *n* = 2281 (41.9 %)	Increased WTC *n* = 482 (8.9 %)	Decreased WTC *n* = 475 (8.7 %)	Level of significance*
	Mean ± SD, *n* (%)	Mean ± SD, *n* (%)	Mean ± SD, *n* (%)	Mean ± SD, *n* (%)	Mean ± SD, *n* (%)	*p*
Age	49.60 ± 9.32	49.17 ± 9.62	50.12 ± 8.94	49.89 ± 9.48	48.79 ± 9.43	<.0013
Sex						<.0001
Women	3075 (56.53)	992 (45.05)	1563 (68.52)	263 (54.56)	257 (54.11)	
Men	2365 (43.47)	1210 (54.95)	718 (31.48)	219 (45.44)	218 (45.89)	
Socio-economic status						<.0001
Unskilled manual workers	1155 (21.70)	103 (4.80)	506 (22.56)	80 (17.06)	65 (13.95)	
Skilled manual workers	1648 (30.96)	164 (7.64)	534 (23.84)	82 (17.48)	82 (17.60)	
Assistant non-manual employees	754 (14.16)	316 (14.71)	286 (12.77)	70 (14.93)	82 (17.60)	
Intermediate non-manual employees	862 (16.19)	679 (31.61)	702 (31.34)	137 (29.21)	130 (27.90)	
Professionals and upper-level executives	754 (14.16)	776 (36.13)	198 (8.84)	94 (20.04)	87 (18.67)	
Self-employed and others	150 (2.82)	110 (5.12)	14 (0.63)	6 (1.28)	20 (4.29)	
Working hours						<.0001
<35 h/week	861 (16.12)	273 (12.60)	442 (19.75)	59 (12.37)	87 (19.00)	
36–40 h/week	2055 (38.48)	644 (29.72)	1062 (47.45)	200 (41.93)	149 (32.53)	
41–45 h/week	1608 (30.11)	771 (35.58)	535 (23.91)	155 (32.49)	147 (32.10)	
46–50 h/week	562 (10.52)	325 (15.00)	139 (6.21)	44 (9.22)	54 (11.79)	
51–55 h/week	132 (2.47)	80 (3.69)	30 (1.34)	11 (2.31)	11 (2.40)	
56–60 h/week	65 (1.22)	44 (2.03)	13 (0.58)	4 (0.84)	4 (0.87)	
>60 h/week	57 (1.07)	30 (1.38)	17 (0.76)	4 (0.84)	6 (1.31)	
Shift work						<.0001
Day time/evening work	4166 (77.54)	957 (45.38)	1138 (52.15)	247 (53.35)	239 (52.88)	
Night work	100 (1.86)	2 (0.09)	91 (4.05)	3 (0.63)	4 (0.85)	
Shift work	379 (7.05)	35 (1.60)	298 (13.26)	24 (5.05)	22 (4.68)	
Roster work	324 (6.03)	31 (1.42)	243 (10.81)	29 (6.11)	21 (4.47)	
Non-regulated	264 (4.91)	186 (8.53)	50 (2.23)	15 (3.16)	13 (2.77)	
Other	140 (2.61)	1876 (86.02)	1501 (66.80)	392 (82.53)	397 (84.47)	
Got new manager	2581 (49.58)	51 (2.34)	64 (2.85)	12 (2.53)	13 (2.77)	<.0001
Changed position						<.0001
Higher	691 (12.94)	376 (17.42)	160 (7.14)	92 (19.37)	63 (13.49)	
Unchanged	4478 (83.84)	1717 (79.53)	2019 (90.13)	362 (76.21)	380 (81.37)	
Lower	172 (3.22)	66 (3.06)	61 (2.72)	21 (4.42)	24 (5.14)	
Experienced WHI 2010	1263 (23.43)	440 (20.14)	591 (26.19)	124 (25.94)	108 (22.93)	<.0001
Experienced WHI 2012	1021 (18.96)	345 (15.76)	478 (21.25)	81 (16.88)	117 (25.00)	<.0001

*p value for GLM and chi-square test

Experiences of WTC were rather stable over the 2-year time (*r* = 0.78). Only about 9 % experienced a decrease or increase in WTC. However, while men mostly reported stable high WTC, most of the women reported low WTC at both time points. Also, socio-economic status differed between WTC categories; around 67 % of professionals and upper-level executives, but only 14 % of the unskilled workers reported having stable high WTC. Further, WTC was positively associated with a higher number of working hours, those with the longest working hours reported most often stable high influence over their working time. Also, shift work (including night work and roster work) was associated with WTC, mostly reporting stable low WTC. Changes in working conditions were not related to major changes in WTC.

Tables 4 and 5 present results from the logistic regression analyses separately for women and men, with results being similar for both groups. In the unadjusted model, both women and men with decreased WTC had higher odds of experiencing WHI than those with stable high WTC. For women, also stable low WTC was related to WHI. After controlling for having children living at home, marital status and socio-economic status (Model 1), both women and men with stable low or decreased WTC showed an excessed odds ratio of WHI. When additionally controlling for work demands, decision authority, working hours and shift work (Model 2), the odds ratio for decreased WTC increased somewhat for both women and men, but men with stable low WTC no longer showed an increased odds for WHI. Changes in work position or the work situation during the past 2 years (Model 3) did not explain the relationship between changes in work time control and WHI. Further controlling for baseline WHI did not change the results much (Model 4). Still, a decreasing WTC was related to higher odds for WHI for both women and men, and while men with stable low WTC had no increased odds for WHI, women with a stable low WTC reported an increased odds ratio for WHI also when controlling for baseline WHI. However, analyses of the additive interaction effect (in the fully adjusted model) between sex and change in WTC on WHI did not reveal any statistically significant difference in the relationship depending on sex. When analysing men and women together in the fully adjusted model (Model 4), both stable low (OR = 1.46; 95 % CI 1.16–1.84) and decreased WTC (OR = 2.14; 95 % CI 1.56–2.93) increased the odds for WHI.

**Table 4:** Results of the logistic regression models for women (*n* = 3075), presented in odds ratios with 95 % confidence intervals of experiencing work–home interference (WHI)

	Cases of WHI	Model 0 uncontrolled	Model 1 adjusted for marital and parental status, and SES	Model 2 additionally adjusted for job demand and control, weekly working hour and shift work	Model 3 additionally adjusted for changes at work^a^	Model 4 additionally adjusted for baseline WHI
Change in WTC						
Stable high	138 (17.8)	Ref	Ref	Ref	Ref	Ref
Stable low	354 (22.6)	1.42 (1.16–1.74)	1.96 (1.57–2.47)	1.46 (1.14–1.88)	1.51 (1.16–1.96)	1.50 (1.12–2.01)
Increase	21 (13.7)	0.95 ( 0.66–1.36)	1.16 (0.79–1.68)	0.85 (0.56–1.28)	0.88 (0.58–1.34)	0.84 (0.53–1.32)
Decrease	66 (27.2)	1.75 (1.27–2.42)	2.07 (1.48– 2.90)	1.99 (1.38–2.85)	2.08 (1.43–3.02)	2.21 (1.45–3.36)
R-square		0.007	0.027	0.112	0.114	0.243

*WTC* work–time control, *SES* socio-economic status

^a^Changed work position and having got a new manager

**Table 5:** Results of the logistic regression models for men (*n* = 2365), presented in odds ratios with 95 % confidence intervals of experiencing work–home interference (WHI)

	Cases of WHI, *N* (%)	Model 0 uncontrolled	Model 1 adjusted for marital and parental status, and SES	Model 2 additionally adjusted for job demands and control, weekly working hour and shift work	Model 3 additionally adjusted for changes at work^a^	Model 4 additionally adjusted for baseline WHI
Change in WTC						
Stable high	128 (14.0)	Ref	Ref	Ref	Ref	Ref
Stable low	131 (16.5)	1.21 (0.94–1.56)	1.36 (1.00–1.83)	1.15 (0.81–1.64)	1.11 (0.76–1.60)	1.19 (0.79–1.80)
Increase	23 (12.8)	1.23 (0.83–1.81)	1.38 (0.92–2.07)	1.26 (0.81–1.95)	1.38 (0.88–2.16)	1.35 (0.80–2.27)
Decrease	43 (21.1)	1.73 (1.21–2.48)	1.88 (1.29–2.74)	1.80 (1.18–2.73)	1.73 (1.12–2.69)	1.95 (1.19–3.20)
R-square		0.004	0.010	0.096	0.095	0.232

*WTC* work–time control, *SES* socio-economic status

^a^Changed work position and having got a new manager

## Discussion

The present study, investigating the longitudinal associations between changes in WTC and WHI, showed that stable low WTC and decreased WTC were associated with higher odds of experiencing WHI. Hence, the results are in line with the findings of a systematic review showing that low WTC is associated with WHI [14] and agrees with results from a natural experiment, indicating a positive effect on work–family conflict after the implementation of unregulated working hours [32]. Still, the number of study participants in the intervention group was small (*n* = 325) and results not generalisable to the working population. Generally, the number of longitudinal studies investigating the effects of WTC is small [14], and there are only few studies investigating the effect of changes in WTC on WHI.

The second research question referred to sex differences, and no clear differences regarding the association between changes in WTC and WHI between women and men were found; the interaction effect was non-significant. Previous research has suggested that women in particular may benefit from control over work time due to sex differences in non-work demands [20, 33]. Indeed, a Dutch study showed that time–spatial flexibility reduced working women’s perceptions of WHI, but not men’s [7]. Yet, work–time flexibility has also been suggested to have negative consequences for women, in the way that women may end up engaging in more non-work responsibilities, rather than using the increased time control to fully recover and reduce strain outcomes [22]. Indeed, a report from the European Commission suggests that flexibility in the length of working time seems to have some adverse effects on gender equality since it increases part-time work among women [11]. In contrast to these suggestions, we could not find any statistically significant difference between men and women on the relationship between change in WTC and WHI. Thus, our results indicate that, at least within a Swedish context, both men and women may benefit from increased control over their working hours. However, in contrast to e.g. the Netherlands, Sweden is a country with a high number of full-time working women and which guarantees subsidised day care to all children above 1 year of age. Another explanation of our findings may relate to the fact that Sweden is a country with high equality between women and men, with increasing number of men sharing the responsibility for household demands.

Additionally, our results suggest that a decrease in WTC has a stronger effect on WHI than a stable low WTC. Indeed, individuals having a low control over their working time may arrange their lives accordingly, whereas those who had a greater influence on their working time but experienced a decrease in control may experience some difficulties before making these arrangements.

Another interesting finding was that WTC seems to be generally stable; less than one fifth reported a change in WTC. This might indicate that WTC is strongly associated with educational level and occupation/profession, and thus factors not changing dramatically during working life. Also, changes in relation to work (new position and/or new manager) were unrelated to changes in WTC, indicating that changes primarily happen within the same socio-economic group and/or occupation. Indeed, most of the study participants had the same job position at both times of measurement (84 %). Also, promotions are often related to increased demands, which may reduce possible positive effects of more WTC. Lastly, WTC is highly inherent to the occupation and depends largely on organisational policies which, in turn, diminishes the influence of the new manager. A high number of employees reported little influence over their working hours. Also, women reported considerably more often than men a stable low WTC (51 vs. 30 %). This is in line with findings reported by the European Commission showing that women are generally more likely to have fixed finishing and starting times than men (68 % of women vs. 58 % of men) [34]. This difference might be partly explained by the fact that women more often work in the public sector and in jobs which require more or less fixed working hours, as in healthcare or education [28].

Also, the perception of WTC and its consequences may be influenced by both need for WTC and opportunities for social support. For individuals with a low need for WTC, e.g. no need to adapt work time to the scheduling of child-care, low WTC is a problem to a much lesser extent than for a single parent with small children and no social support. Unfortunately, we had no possibility to control for social support outside work, although this could be a possible confounder influencing the relationship between changes in WTC and WHI.

Our study adds to the knowledge on the potential effects of changes in WTC on WHI. However, some shortcomings should be acknowledged. First, the rather small groups of workers who report increased or decreased control over their working hours limit statistical power. Yet, the results indicated that decreasing and stable low WTC might have an influence on WHI. Second, despite the longitudinal study design, no firm conclusions about causality can be drawn. Other changes at the workplace may foster the contemporary improvement in WTC and WHI. Although we adjusted for a change in position or getting a new manager, we cannot exclude that other important changes have influenced the results. Still, controlling for a number of possible confounders did not alter the results much, and so there is reason to consider an increased WTC as a promising tool for improving employees work–life balance.

Of course, the 2-year time lag can be considered a weakness. Two years may be too long to detect effects especially as work–family conflict may arise on a day-to-day level. Still, the 2-year time lag is similar to what other large occupational cohort studies (e.g. the Finish Public Sector study) use. Also, analyses revealed some differences between those who had missing data at one or several study variables and those who had full information on all study variables. Those with missing data on one or several study variables were in average younger, had lower socio-economic position and worked more often shift-work (*p* < 0.01). However, no differences in sex or weekly working hours were observed. Also, participants with low WTC at baseline did answer the follow-up questionnaire to the same extent as participants with high baseline WTC. The study group was relatively old (mean age was 49 years), which suggests that employees with small children are underrepresented in the sample. This might also explain the general decrease in WHI over the 2 years. As a consequence, the prevalence of WHI might have been underestimated. Additionally, relying solely on self-reports may involve social desirability and the ordering and wording of items may bias participants’ ratings. Using only questionnaire data may also result in common method bias. The feeling of being able to influence work–time aspects may be contaminated by other work-related variables, e.g. the relation to the supervisor. Also, individuals who are satisfied with their working hours might report more WTC than the unsatisfied, although they have objectively the same control over working time. However, a comparison between survey-reported ratings of employee control and qualitative data on existing practices supports self-reports as a valid measure [35, 36]. Furthermore, the construct of WHI is adequately assessed through self-reports in questionnaires, and objective measures are not available. Supported by finding by Nijp [14] who reported moderately strong evidence that WTC promotes better work–life balance, we are confident that our results are not explained by reporting issues. Further, we studied only availability of WTC and not the actual use of it. Moreover, we used a composite measure of WTC which covers rather two different aspects of flexibility, i.e. control over daily hours and control over time off [6]. Future research should investigate the impact of the different sub-dimensions of WTC and their impact on different outcomes. For example, the possibility to take breaks when wanted or needed may be related primarily to recovery, whereas the possibility to adjust starting and finishing times may be more strongly related to work–life balance.

In summary, our findings based on a large representative cohort of Swedish employees indicate that low and decreased control over working hours may have negative effects on WHI for both women and men. The practical implication of this observation is that increased WTC can reduce the conflict between work and non-work life, which in turn could improve well-being and decrease work stress-related health problems.
